# Obesity, Lifestyle Habits and Nutrients in Relation to Oral Ulcers: A Comprehensive Mendelian Randomization Study

**DOI:** 10.1002/hsr2.72829

**Published:** 2026-07-16

**Authors:** Shuai Kang, Junkai Cao, Yu Hou, Jie Geng, Huanhuan Yuan, Quan Shi, Shuai Shan, Tong Zhang, Yi Jiang

**Affiliations:** ^1^ Institute of Stomatology Chinese People's Liberation Army No.989 hospital Luoyang China; ^2^ Institute of Stomatology, First Medical Center Chinese People's Liberation Army General Hospital Beijing China; ^3^ Institute of Basic Medical Sciences Shanxi Medical University Jinzhong China

**Keywords:** genetic epidemiology, immunity, lifestyle habits, mediation analysis, Mendelian randomization analysis, oral ulcer

## Abstract

**Background and Aims:**

Previous studies demonstrated a potential association between obesity, lifestyle habits, nutrients and mouth ulcers. However, this relationship remains inadequately examined. The current study seeks to further explore the associations between exposures and mouth ulcers, employing a Mendelian randomization (MR) approach to evaluate causality.

**Methods:**

Genetic variants identified in genome‐wide association studies (GWAS) of eight exposures were employed as instrumental variables. This study utilized Univariable MR (UVMR), multivariable MR (MVMR), and mediation MR approaches.

**Results:**

The study uncovered a causal relationship between 6 exposures and mouth ulcers via UVMR and Meta‐UVMR. After adjusting for confounding factors through MVMR, there was still a significant positive correlation between alcohol consumption (odds ratios (95% confidence intervals): OR (95%CI) = 1.0184 (1.0049,1.0321), FDR = 0.02), body mass index (BMI) (OR (95%CI) = 1.0104 (1.0050,1.0157), FDR < 0.001) are positively correlated with mouth ulcers, while there was a negative correlation between sleep duration (OR (95%CI) = 0.9773 (0.9648,0.9899), FDR = 0.002) and mouth ulcers. Mediation analysis indicated that 12 mediators may play a mediating role in the pathogenesis of alcohol consumption‐mouth ulcers, 2 mediators in BMI‐mouth ulcers, and 5 mediators in sleep duration‐mouth ulcers.

**Conclusion:**

The causal relationship between the three exposures (alcohol consumption, BMI, and sleep duration) and mouth ulcers was analyzed, partially mediated by circulating cytokines, metabolites, and plasma proteomes.

## 1 Introduction

Mouth ulcers are prevalent in humans, affecting up to 25% of the general population, with a higher incidence among young adults [1]. Mouth ulcers often cause severe pain that can impact eating, swallowing, speaking, and psychosocial functioning [2]. During the disease progression, severe injuries may occur, accompanied with fever and even extensive ulcers in some poor circumstances [3]. Mouth ulcers are regarded as an early indicator for certain cancers, such as oral squamous cell carcinoma (OSCC) [4, 5]. While certain cases are attributable to local stimuli such as mechanical or physical factors, the precise etiology of mouth ulcers remains obscure, which is believed that numerous factors, including genetic susceptibility, immune factors, nutritional deficiency, and lifestyle, may contribute to the development of the disease [6]. The challenge of treating and preventing the disease is compounded by unknown etiologies; therefore, identifying the risk factors for mouth ulcers is vital in developing effective strategies for monitoring, prevention, and disease control.

Obesity, lifestyle habits, and nutrients have been proved to significantly influence the occurrence of mouth ulcers and are crucial for disease prevention. Sleep is an essential process crucial for regulating the body's core biological functions and immune system, and studies have indicated its significant role in maintaining oral health as well [7, 8]. Research suggests that individuals with higher Body Mass Index (BMI) are more susceptible to dental caries and periodontal disease [9]. Vitamin C and D are widely recognized nutrients, and a deficiency in these vitamins may potentially contribute to the development or exacerbation of oral ulcers, according to certain studies [10, 11]. Exercise/sedentary behaviors influence immune regulation, acting as protective or risk factors for various immune‐related diseases, including periodontitis and gastric ulcers [12, 13, 14]. Smoking and alcohol consumption, widespread unhealthy habits, not only directly cause oral diseases through local stimulation but also affect the overall immune system, potentially triggering immune‐related ailments [15, 16]. The impact of these factors on the incidence of mouth ulcers remains unclear, posing challenges in developing effective treatment and prevention strategies. Further investigation into the causal relationships between obesity, lifestyle habits, nutrients and mouth ulcers is essential.

Observational studies often face limitations such as small sample sizes and inadequate control of significant confounders. However, Mendelian randomization (MR) analysis uses genetic variation as instrumental variables (IVs) to explore the causality between exposure factors and outcome events and is regarded as a naturally occurring randomized controlled trial within the population, offering advantages in controlling for confounders and reverse causality [17, 18, 19].

In this MR study, we investigated the influence of various factors on oral ulcer through Univariable MR (UVMR), Meta‐UVMR, Multivariate MR (MVMR), and mediation MR, in order to understand the root causes and influencing factors of oral ulcers, thereby providing strategies for the prevention and treatment.

## 2 Methods

### 2.1 Study Design

**Figure 1:**
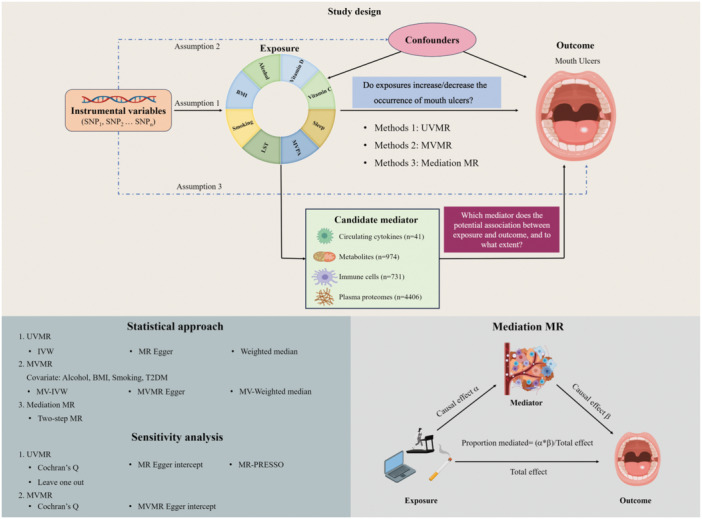
Overview of the study design. This MR study comprised two distinct analysis phases. Initially, we evaluated the causal association between exposures and mouth ulcers phenotype by employing UVMR, while adjusting for potential confounders through MVMR. In the second phase, we conducted a screening of 6152 candidate mediators pertaining to circulating cytokines, metabolites, immune cells and plasma proteomes. Furthermore, we quantified the mediating impact of each individual mediator using a two‐step MR approach. BMI, Body Mass Index; IVW, Inverse Variance Weighted; LST, Leisure Screen Time; MVPA, Moderate‐to‐Vigorous intensity Physical Activity; MVMR, Multivariable Mendelian randomization; SNPs, single nucleotide polymorphisms; UVMR, Univariable Mendelian randomization.

Since all the summary‐level genome‐wide association studies (GWAS) data utilized in the analysis were publicly accessible, this study was exempt from ethical review. Figure 1 displayed a flowchart illustrating the study's methodology. In the flowchart, eight distinct factors were selected as exposures, with mouth ulcers being as the designated outcome. To investigate potential causal relationships between exposures and mouth ulcers, we employed a UVMR analysis. Single nucleotide polymorphisms (SNPs) as IVs for Mendelian randomization must satisfy the following assumptions: (1) SNPs must be strongly associated with the exposures; (2) SNPs must affect the outcomes only through the exposures; (3) No potential known confounders that caused both the SNPs and exposures/outcomes [20, 21]. In our study, alcohol consumption, smoking, BMI, and type 2 diabetes mellitus (T2DM) were identified as confounders for the relationship between exposures and mouth ulcers. Multivariate MR analysis was conducted to delineate the direct causal relationship between exposures and mouth ulcers while adjusting for those confounders. Additionally, a Mediation MR analysis was carried out to assess the potential mediating roles between exposures and mouth ulcers. This report followed the Strengthening the Reporting of Observational Studies in Epidemiology using Mendelian randomization (STROBE‐MR) statement and was not pre‐registered [22].

### 2.2 GWAS Data Source

#### 2.2.1 Data Source of Exposure

We utilized leisure screen time (LST) as a proxy for sedentary behavior and moderate‐to‐vigorous intensity physical activity (MVPA) as an indicator of exercise. Genetic variants related to LST (*n* = 526,725) and MVPA (*n* = 608,595) were obtained from a GWAS meta‐analysis comprising 51 European population studies [23]. Both phenotypes were assessed using self‐reported questionnaire data. Summary statistics on sleep duration were derived from GWAS using data from the UK Biobank (*n* = 446,166) [24]. We assessed smoking heaviness and alcohol consumption, using cigarettes per day (*n* = 377,334) and drinks per week (*n* = 941,280) as respective indicators [25]. Comprehensive summary statistics on circulating vitamin C concentration were provided by a recent GWAS. This genome‐wide association study included 52,018 individuals of European descent from four separate studies [26]. Summary statistics for circulating vitamin D concentration were derived from a GWAS performed by the UK Biobank (UKB), involving 496,946 Europeans aged between 40 and 69 years, representing a vast population‐based cohort of European descent [27].

#### 2.2.2 Data Source of Outcome and Confounders

We obtained pooled data on the association between SNPs and mouth ulcers by extracting information from two GWAS studies. The discovery data concerning mouth ulcers were sourced from deposits containing summary statistics of genome‐wide analysis for dental endpoints in the GLIDE consortium, and the UK Biobank, as well as a multi‐trait analysis combining data from both GLIDE and UKB (*n* = 461,031) [28]. The replication date was derived from the UKB, which includes 461,106 Europeans and 9,851,866 genetic variants [29]. Information regarding mouth ulcers was gathered retrospectively through a questionnaire, which asked participants to provide their responses.

Furthermore, for MVMR analysis, we meticulously selected SNPs associated with BMI (IEU: ukb‐b‐19,953), T2DM [30], cigarettes per day [25], and drinks per week [25] based on published GWAS summary statistics.

#### 2.2.3 Data Source of Candidate Mediators

In the mediation analysis, we included the data on 41 circulating cytokines, 974 metabolites, 731 immune cells and 4406 plasma proteomes. The circulating cytokines data were sourced from a study that investigated genomic variant associations with 41 cytokines and growth factors across 8293 individuals, integrating results from both the Cardiovascular Risk in Young Finns Study and the FinRisk Survey [31]. Immune cells data were derived from the GWAS catalog, which boasts accession numbers spanning from GCST0001391 to GCST0002121 and provides comprehensive summary statistics from GWAS related to each immunological trait [32]. Summary statistics for blood metabolites were obtained from comprehensive surveys conducted by Shin et al. (*n* = 7824) [33], Roederer et al. (*n* = 669) [34], Kettunen et al. (*n* = 24,925) [35] and Julkunen et al. (*n* = 118,461) [36]. For plasma proteome, the summary statistics were derived from a comprehensive survey conducted by Sun et al. (*n* = 3301) [37], Folkersen et al. (*n* = 2639) [38] and Suhre et al. (*n* = 997) [39]. Detailed information on data sources was presented in Table 1.

**Table 1:** Overview of the GWAS data used in the study.

Phenotype	ID	Participants	Ancestry	Data source	Author/Year
Alcohol consumption	GCST9007461	941,280	European	GSCAN	Liu/2019
Smoking heaviness	GCST9007459	377,334	European	GSCAN	Liu/2019
BMI	ukb‐b‐19953	461,460	European	MRC‐IEU	Ben Elsworth/2018
LST	GCST90104339	526,725	European	UKB	Wang/2022
MVPA	GCST90104341	608,595	European	UKB	Wang/2022
Sleep duration	GCST9007561	446,166	European	UKB	Dashti/2019
Vitamin C	GCST011816	52,018	European	FinnGen, PEIC	Zheng/2020
Vitamin D	GCST90000614	417,580	European	UKB	Revez/2020
Discovery	GCST008304	461,031	European	GLIDE and UKB	Shungin/2019
Replication	GCST007839	461,106	European	UKB	Dudding/2019
Smoking heaviness	GCST9007459	377,334	European	GSCAN	Liu/2019
Alcohol consumption	GCST9007461	941,280	European	GSCAN	Liu/2019
BMI	ukb‐b‐19953	461,460	European	MRC‐IEU	Ben Elsworth/2018
T2DM	GCST90018706	European (*N* = 490,089) East Asian (*N* = 177,415)	European/East Asian	UKB, FinnGen, BBJ	Sakaue/2021
Circulating cytokines (*n* = 41)	GCST004020 ‐ GCST004060	8293	European	YFS and FINRISK	Ahola‐Olli/2016
Metabolites (*n* = 452)	ieu‐met‐a	7824	European	MRC‐IEU	Shin/2014
Metabolites (*n* = 150)	ieu‐met‐b	669	European	MRC‐IEU	Roederer/2015
Metabolites (*n* = 123)	ieu‐met‐c	24,925	European	MRC‐IEU	Kettunen/2016
Metabolites (*n* = 249)	ieu‐met‐d	118,461	European	MRC‐IEU	Nightingale Health/2020
Immune cells (*n* = 731)	GCST90001391 – GCST90002121	3669	European	MRC‐IEU	Orrù/2020
Plasma proteomes (*n* = 3282)	ieu‐prot‐a	3301	European	MRC‐IEU	Sun/2018
Plasma proteomes (*n* = 83)	ieu‐prot‐b	2639	European	MRC‐IEU	Folkersen/2017
Plasma proteomes (*n* = 1124)	ieu‐prot‐c	997	European	MRC‐IEU	Suhre/2017

Abbreviations: BBJ, Biobank Japan Project; BMI, Body Mass Index; EPIC, European Prospective Investigation into Cancer and Nutrition; GSCAN, GWAS and Sequencing Consortium of Alcohol and Nicotine use; LST, Leisure Screen Time; MRC‐IEU, Medical Research Council‐ Integrative Epidemiology Unit; MVPA, Moderate‐to‐Vigorous intensity Physical Activity; T2DM, Type 2 Diabetes Mellitus; UKB, UK Biobank; YFS, Young Finns Study.

### 2.3 Selection of IVs

To satisfy the initial core MR assumption, we selected genetic instruments linked to exposures with genome‐wide significance set at *P* < 5 × e^−8^. Based on the European 1000 Genomes dataset, we performed a linkage disequilibrium (LD) analysis (*r* ^2^ < 0.001, within window size = 10 Mb) to strengthen the independence of the instruments. After harmonizing, palindromic SNPs were removed without replacing missing SNPs with proxy variants. Moreover, we assessed the strength of each remaining SNP solely through the *F*‐statistic (*F* = *β* ^2^/SE^2^, *β* is the estimated effect of the SNP on the exposure and SE is its standard error), excluding SNPs with an *F*‐statistic <10. Given the limited number of SNPs identified in certain media during the IVs selecting period, a higher threshold (*P* < 1 × e^−5^) was employed in the Mediation MR analysis.

### 2.4 Mendelian Randomization Analysis

#### 2.4.1 UVMR and MVMR

UVMR, Meta‐UVMR, and MVMR are our pre‐designated primary analyses. In UVMR, a primary analysis was conducted utilizing the Inverse Variance Weighted (IVW) test to evaluate causality. This test, incorporating multiplicative random effects, was deemed the most effective approach for deriving causal effect estimates, while also accounting for the heterogeneity observed in these estimates [40]. We assessed the bias and type 1 error rate resulting from sample overlap via using the online calculator (https://sb452.shinyapps.io/overlap/). After evaluation, the type I error rate consistently maintained at 0.05 across all analyses, even though the samples were completely overlapping [41]. Additionally, we utilized two other methods to further validate our findings, including MR‐Egger and weighted median. To ensure the robustness of our findings, we conducted a meta‐analysis combining the results from two separate mouth ulcers datasets. In MVMR, the primary analysis was conducted using MV‐IVW, which effectively accounted for the correlation among multiple exposures. To bolster and enhance reliability, MVMR‐Egger and MV‐weighted median analyses were also conducted.

#### 2.4.2 Mediation MR

As a secondary, exploratory aim, we performed a mediation analysis to identify potential biological pathways linking the identified causal exposures to mouth ulcers. Results from the mediation analysis should be interpreted as hypothesis‐generating and require validation in independent cohorts. In the mediation analysis, the indirect effects of each medium were determined via using a two‐step MR approach. Initially, the causal effect α of exposures on a hypothesized mediator was estimated. Subsequently, the causal effect β of the mediators on the outcome was established.

The mediation effect was determined by the equation: Mediation effect = α*β. The proportion of the mediation effect was calculated via dividing the total causal effect of exposures on mouth ulcers by the mediation effect, confidence intervals were estimated by the delta method [42]. The calculation process was described in detail in Supplementary Figure 1.

### 2.5 Sensitivity Analyses

Several sensitivity analyses were conducted to assess the effect of potential pleiotropy and verify the second and third MR assumptions. Cochran's *Q*, MR‐Egger, MR‐PRESSO and leave‐one‐out analysis were performed to verify the foundational assumptions for UVMR. Additionally, Cochran's *Q* and MVMR‐Egger tests were performed for MVMR. The Cochran's *Q* test (*p* < 0.05 indicates significant heterogeneity) was used to evaluate the heterogeneity of the individual SNP effects and detect the presence of pleiotropy [43]. The MR/MVMR‐Egger intercept test was performed to assess directional pleiotropy, with an intercept significantly differing from zero (*p* < 0.05), suggesting the presence of overall directional pleiotropy [44]. The MR‐PRESSO method was employed to test for general level pleiotropy and outliers by analyzing the sum of global and SNP‐specific effects [44]. Moreover, the leave‐one‐out analysis aimed to explore the influence of each genetic variation on the outcomes.

### 2.6 Statistical Analyses

All MR estimates were presented as odds ratios (ORs) along with 95% confidence intervals (CIs). In consideration of the potential increase in cumulative Type I error across multiple comparisons, the Benjamini–Hochberg method was employed to control the false discovery rate (FDR) effectively and adjusted for multiple testing [45]. All reported *p* values are from two‐sided hypothesis tests, and a significance level of *α* = 0.05 was set a priori for the primary analyzes. An FDR significance threshold of < 0.05 indicated a significant association, while a *p* < 0.05 but FDR > 0.05 suggested a suggestive association. All of the aforementioned processes were executed by R 4.4.0. The R Package Two‐Sample MR (version 0.6.4), MVMR (version 0.4), MRPRESSO (version 1.0) and Mendelian Randomization (version 0.10.0) were utilized for MR and sensitivity analyses.

## 3 Results

### 3.1 Univariable MR

**Figure 2:**
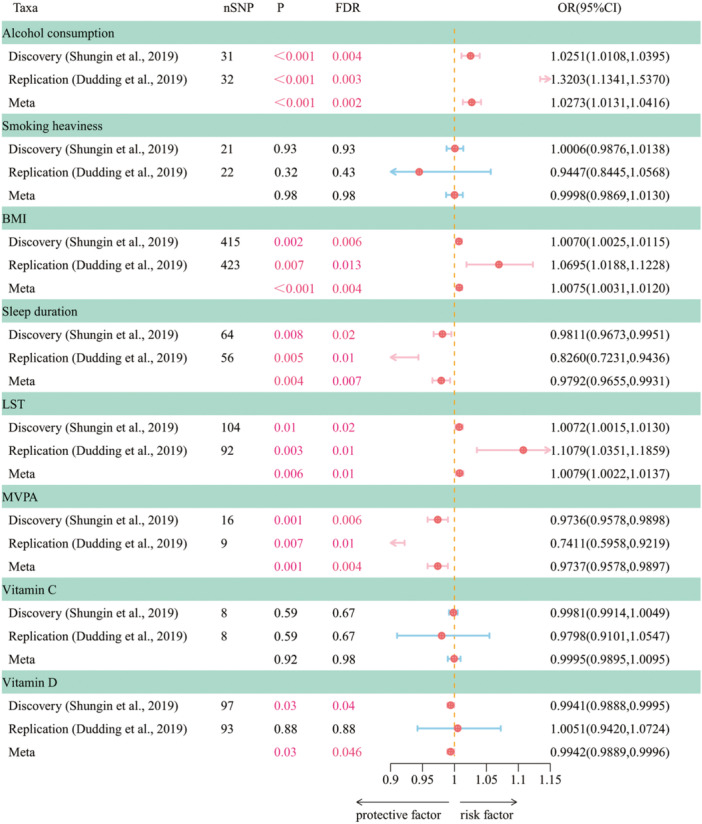
UVMR estimates for the causal associations between exposures and mouth ulcers via IVW method. BMI, Body Mass Index; CI, confidence intervals; FDR, false discovery rate; IVW, Inverse Variance Weighted; LST, Leisure Screen Time; MVPA, Moderate‐to‐Vigorous intensity Physical Activity; OR, odds ratios; SNPs, single nucleotide polymorphisms; UVMR, Univariable Mendelian randomization.

The descriptive statistics of the study phenotypes related to exposures and outcomes included in the GWAS are shown in Supplementary S1. All selected SNPs had F‐statistics > 10, indicating sufficient instrument strength. UVMR analyses suggested causal associations for six exposures with mouth ulcers (Figure 2).

In the primary IVW analysis, genetically predicted alcohol consumption was positively associated with mouth ulcers in both discovery (OR (95%CI) = 1.0251 (1.0108–1.0395), FDR = 0.004) and replication datasets (OR (95%CI) = 1.3203 (1.1341–1.5370), FDR = 0.003), with consistent results in the Meta‐analysis (OR (95%CI) = 1.0273 (1.0131–1.0416), FDR = 0.002).

Similarly, BMI showed significant positive associations (Discovery: OR (95%CI) = 1.0070 (1.0025, 1.0115), FDR = 0.006; Replication: OR (95%CI) = 1.0695 (1.0188, 1.1228), FDR = 0.013; Meta‐analysis: OR = OR (95%CI) = 1.0075 (1.0031, 1.0120), FDR = 0.004). Sleep duration showed consistent protective effects (Discovery: OR (95%CI) = 0.9811 (0.9673, 0.9951), FDR = 0.02; Replication: OR (95%CI) = 0.8260 (0.7231, 0.9436), FDR = 0.01; Meta‐analysis: OR (95%CI) = 0.9792 (0.9655, 0.9931), FDR = 0.007).

For other lifestyle factors, LST was positively associated with mouth ulcers (Meta‐analysis: OR (95%CI) = 1.0079 (1.0022, 1.0137), FDR = 0.01), while MVPA (Meta‐analysis: OR (95%CI) = 0.9737 (0.9578, 0.9897), FDR = 0.004) and Vitamin D (Meta‐analysis: OR (95%CI) = 0.9942 (0.9889, 0.9996), FDR = 0.046) showed inverse associations. The detailed results of these three MR methods were displayed in Supplementary S2, where tendencies from the MR Egger and Weighted Median analyses aligned with the IVW estimates, although not all the results in these analyses reached significant differences.

In the majority of sensitivity analyses conducted, a consistent directionality of causal estimates was observed, as indicated in Supplementary Table 1. The Cochran's *Q* test revealed some heterogeneity in the causal relationship between BMI (Discovery and Replication) and mouth ulcer, and horizontal pleiotropy between LST (Replication) and mouth ulcer was detected via the MR‐Egger intercept test. The MR‐PRESSO test identified some outlier SNPs (Supplementary S3), then we removed the outliers and re‐analyzed. The leave‐one‐out test showed that there was no obvious bias in the selected SNPs (Supplementary Figure 2).

Further information and visual depictions of the data analysis, which comprises scatter plots pertaining to the pleiotropy analysis, and funnel plots (Supplementary Figures 3,4).

### 3.2 Multivariate MR

**Figure 3:**
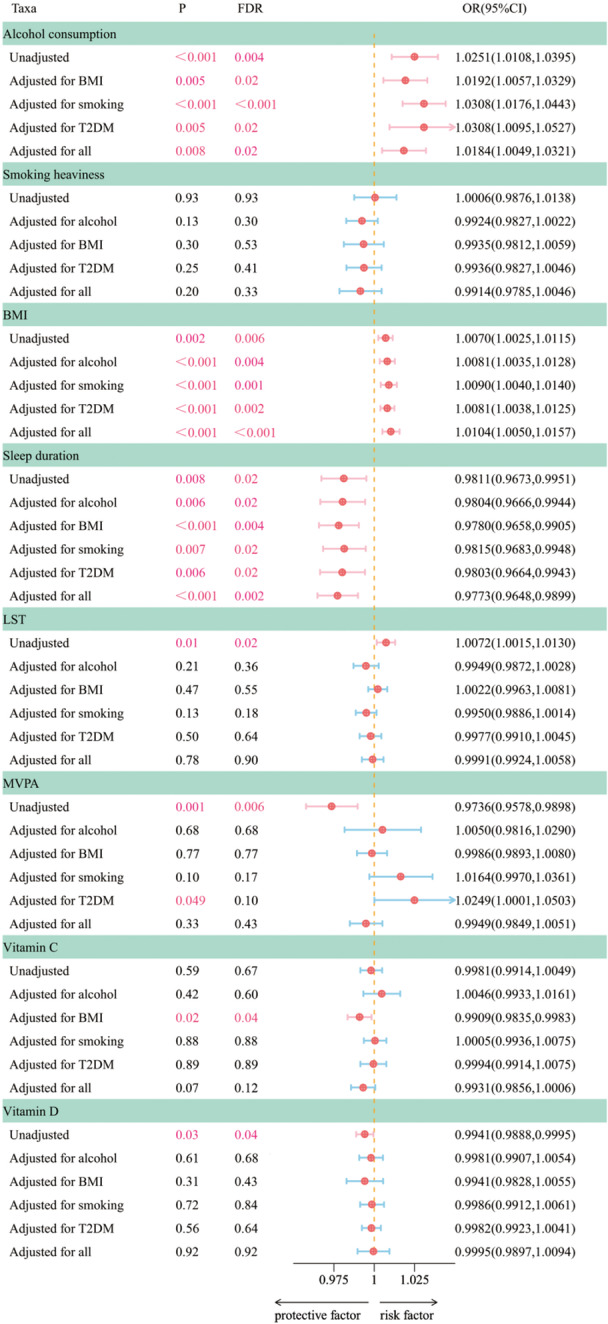
MVMR estimates for the causal associations between exposures and mouth ulcers via IVW method. BMI, Body Mass Index; CI, confidence intervals; FDR, false discovery rate; IVW, Inverse Variance Weighted; LST, Leisure Screen Time; MVPA, Moderate‐to‐Vigorous intensity Physical Activity; MVMR, Multivariable Mendelian randomization; OR, odds ratios; SNPs, single nucleotide polymorphisms.

We also performed MVMR for exposures shown in Figure 3. After adjusting the factors of alcohol, smoking, BMI and T2DM, only three exposures remained statistical significance consistently of MV‐IVW methods. (Alcohol consumption: OR (95%CI) = 1.0184 (1.0049,1.0321), FDR = 0.02; BMI: OR (95%CI) = 1.0104 (1.0050,1.0157), FDR < 0.001; Sleep duration: OR (95%CI) = 0.9773 (0.9648,0.9899), FDR = 0.002). However, the other three lifestyle habits were attenuated after the adjustment of these confounders (LST: OR (95%CI) = 0.9991 (0.9924,1.0058), FDR = 0.90; MVPA: OR (95%CI) = 0.9949 (0.9849,1.0051), FDR = 0.43; Vitamin D: OR (95%CI) = 0.9995 (0.9897,1.0094), FDR = 0.92). Detailed data of these findings are present in Supplementary S4.

In the sensitivity analysis, MVMR‐Egger regression within the MVMR framework did not reveal any horizontal pleiotropy; however, Cochran's *Q* test indicated some heterogeneity (Supplementary Table 2).

### 3.3 Mediation MR

**Figure 4:**
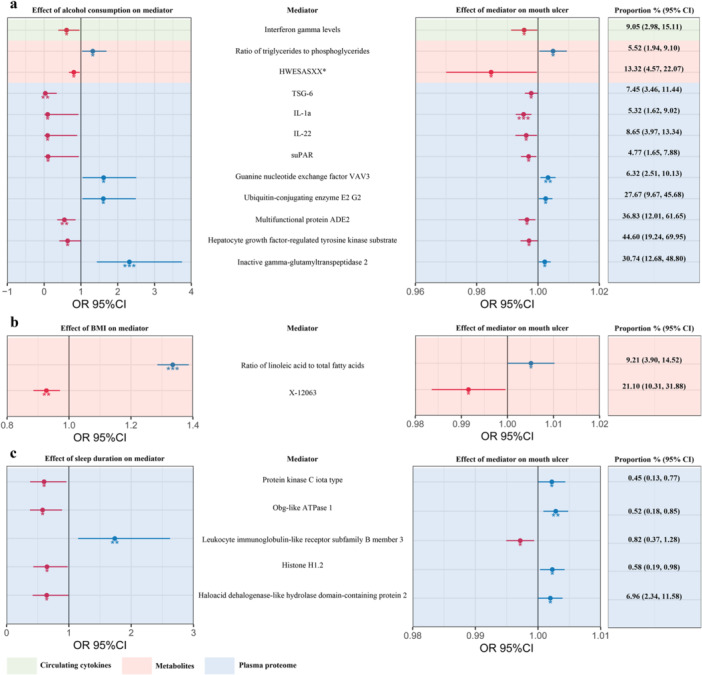
Mediating role of each mediator in the causal association between the exposures and mouth ulcers. (a) Mediation MR of alcohol consumption on mouth ulcers; (b) mediation MR of BMI on mouth ulcers; (c) mediation MR of sleep duration on mouth ulcers. BMI, Body Mass Index; CI, confidence intervals; MR Mendelian randomization; OR, odds ratios.

For exposures with significant UVMR and MVMR findings, we performed mediation analysis. From 6152 candidates, 19 passed the predefined selection criteria and were evaluated as potential mediators. In the mediation analysis assessing the impact of alcohol consumption (Figures 4a), 1 circulating cytokine, 2 metabolites and 9 plasma proteomes were identified as mediators for mouth ulcers, including Interferon gamma levels (mediation proportion, 9.05%), Ratio of triglycerides to phosphoglycerides (5.52%), HWWESASXX* (13.32%), TSG‐6 (7.45%), IL‐1a (5.32%), IL‐22 (8.65%), suPAR (4.77%), Guanine nucleotide exchange factor VAV3 (6.32%), Ubiquitin‐conjugating enzyme E2 G2 (27,67%), Multifunctional protein ADE2 (36.83%), Hepatocyte growth factor‐regulated tyrosine kinase substrate (44.60%) and Inactive gamma‐glutamyl transpeptidase (30.74%). Each mediator contributed more than 5% to the total effect of alcohol consumption on mouth ulcers, except for suPAR. For BMI (Figures 4b), two metabolites were identified as mediators for mouth ulcers, including the ratio of linoleic acid to total fatty acids (9.21%) and X‐12063 (21.10%); all mediators constituted more than 5% of the total effect. As to sleep duration (Figure 4c), five plasma proteomes were identified as mediators for mouth ulcers, including Protein kinase C iota type (0.45%), Obg‐like ATPase 1 (0.52%), Leukocyte immunoglobulin‐like receptor subfamily B member 3 (0.82%), Histone H1.2 (0.58%) and Haloacid dehalogenase‐like hydrolase domain‐containing protein 2 (6.96%), only 1 mediator accounted for more than 5% of the total effect.

## 4 Discussion

Obesity, lifestyle habits, nutrients, and some other controllable factors, have been proved to significantly influence the oral health and are crucial for disease prevention. In this comprehensive MR study, we identified independent causal associations between three modifiable exposures—alcohol consumption, BMI, and sleep duration—and the risk of mouth ulcers, with evidence of mediation through circulating cytokines, metabolites, and plasma proteomes. This study is pioneering in elaborating the heritability between obesity, lifestyle habits, nutrients and mouth ulcers, highlighting the essential role of those exposures to mouth ulcers. Given these findings, maintaining a healthy lifestyle is critical for both prevention and treatment strategies. And these mediators possess the potential to function as biomarkers for predicting mouth ulcers within specific populations, however, further examination is necessary to ascertain if these mediators can serve as biomarkers or therapeutic targets.

We acknowledge that the observed ORs (e.g., ~1.01 for BMI, ~1.02 for alcohol consumption) are clinically modest. Despite statistical significance, these small relative effects do not support individual‐level clinical prediction. However, given the high population prevalence of these exposures, even modest risk shifts may translate into meaningful absolute burden at the public health level.

Clinically, the common, self‐limiting ulcers examined in this study—typically painful and healing within 1–2 weeks—differ clinically from OSCC‐related ulcers, which are indurated, irregular, and non‐healing [5]. Our GWAS data do not permit direct distinction between these entities, but the very low population prevalence of OSCC relative to benign ulcers means that our Mendelian randomization estimates primarily reflect causal effects on benign ulcer risk.

Thus far, several causes of oral ulceration have been identified, encompassing local mucosal damage as well as a variety of autoimmune and inflammatory disorders [1, 46]. Alcohol consumption contributes to over 60 diseases and ~2.5 million deaths annually [47]. It is also associated with a higher prevalence of oral mucosal lesions, including ulcers and leukokeratosis [48]. Alcohol exerts systemic effects by triggering inflammatory responses and metabolic disturbances, and locally by irritating oral mucosa and potentially contributing to malignant transformation [49, 50]. Also, studies have reported an association between alcohol intake and alterations in circulating cytokines, immune cells, metabolites and plasma proteomes, including IL‐6/8/10/12, TNF‐α, C‐reactive protein, macrophage inflammatory protein, cytotoxic T‐cells, HDL, cholesterol, Low LDL and various plasma proteomes [51, 52, 53]. While the link between obesity and mouth ulcers is not well established, metabolic syndrome has been associated with increased ulcer risk. One case‐control study found that ulcer patients had higher BMI and neck circumference than controls, implying that obesity‐related metabolic and inflammatory factors may play a role, though definitive evidence is lacking [54]. Some studies have demonstrated that good sleep can effectively reduce the risk of mouth ulcers [55]. One potential explanation was that sleep duration could influence oral ulcers through its regulation of immune responses and inflammatory mediators [8, 56]. It has been suggested that sleep deprivation elevates serum concentrations of TNF‐α, IL‐1β, IL‐6, IL‐8, and MCP‐1 [57]. Furthermore, excessive production of TNF‐α, IL‐1β, and IL‐6 has been linked to a heightened risk of developing mouth ulcers [58]. Our MR analysis confirmed that alcohol, BMI, and sleep duration may mediate mouth ulcer risk through cytokines, metabolites, and proteomes. As the first large‐scale screening of such mediators, this study offers potential insights into biomarkers for at‐risk individuals (e.g., heavy drinkers, obese, or sleep‐deprived) and possible therapeutic targets. Nevertheless, these findings are exploratory and need independent replication; the relaxed instrument selection threshold and lack of multiple‐testing correction in this phase mean that the identified mediators require further independent validation before being considered robust.

Through our study, we found that other exposures such as exercise, sedentary behavior, vitamin D, etc, may be related to mouth ulcers, but the current evidence is insufficient. Currently, there are limited studies exploring the correlation between exercise/sedentary behavior and mouth ulcers. However, exercise profoundly modulates immune function by mobilizing cytotoxic cells (e.g., NK cells, CD8 + T‐cells) and innate responders such as monocytes and neutrophils [59]. On the contrary, two studies have indicated that sedentary behavior is linked to a heightened risk of metabolic syndrome, augmented insulin resistance, and raised C‐reactive protein levels [60, 61]. A meta‐analysis suggested a significant association between low vitamin D levels and mouth ulcers [62]. Given Vitamin D's well‐established role in both innate and adaptive immunity, this may explain its observed association with mouth ulcers [63]. Our UVMR and Meta‐UVMR findings aligned with these observations, but the associations disappeared in MVMR after adjusting for smoking, BMI, alcohol, and T2DM, indicating that these four confounders likely account for the apparent effects.

There is no evidence indicating that vitamin C intake has any impact on the development of oral ulcers, aligning with the findings of our study [64]. Interestingly, a cross‐sectional survey had indicated that smokers exhibit a reduced incidence of mouth ulcers compared to non‐smokers [65]. Smoking showed a non‐significant inverse trend in MVMR, a pattern that may be explained by smoking‐induced mucosal keratinization, though this interpretation remains speculative.

To the best of our knowledge, this study represented the inaugural application of UVMR, Meta‐UVMR and MVMR in comprehensively analyzing the causal relationship between obesity, lifestyle habits, nutrients and mouth ulcers. Furthermore, through the examination of potential mediators, we can enhance our comprehension of the fundamental mechanisms and facilitate the advancement of preventative and therapeutic interventions. The application of the MR method aided in minimizing confounding bias and obtaining robust estimates of causal effects. And to ensure the reliability of the results, multiple sensitivity analyses and assessments of IV intensity were conducted.

Inevitably, this study was not without limitations. First, the identification of oral ulcers relies on participants' self‐reported experiences of oral ulcers in the past, rather than clinical diagnoses. Consequently, the GWAS data are unable to differentiate between various types of oral ulcers, potentially introducing a degree of bias. Second, it was advisable to interpret causality with caution, although multiple sensitivity analyses and supplementary test results consistently demonstrated that factors such as weak IVs, horizontal pleiotropy, outliers, and sample overlap did not violate the fundamental MR hypothesis and did not significantly impact our causal estimates. This was because several assumptions inherent in these methods could not be directly validated, and variations in IVs strength and residual horizontal pleiotropy may potentially introduce biases into certain results. Third, this study did not comprehensively capture all mediating pathways. Fourth, the data we used were all from European ancestry, so the results may not be generalizable to populations of other ancestries. Fifth, the modest effect sizes, particularly for BMI and alcohol consumption, have limited clinical utility for individual prediction, though they remain meaningful for population‐level risk assessment. Finally, even when the *F*‐statistic was guaranteed to exceed 10, the explanatory power of the IVs for potential mediating variables remained limited, which can be further explored in the future.

## 5 Conclusion

In conclusion, our results support causal roles for alcohol consumption, BMI, and sleep duration in mouth ulcer risk, with mediation through immune and metabolic pathways. While effect sizes are small at the individual level, their population‐level relevance underscores the importance of lifestyle interventions as part of oral health promotion. Future studies are needed to validate the identified mediators as potential biomarkers or therapeutic targets.

## Author Contributions

**Shuai Kang:** writing – review and editing, conceptualization, methodology, formal analysis, software. **Junkai Cao:** writing – original draft, data curation. **Yu Hou:** writing – review and editing, validation. **Jie Geng:** writing – review and editing, methodology. **Huanhuan Yuan:** writing – review and editing, investigation. **Quan Shi:** writing – review and editing, supervision. **Shuai Shan:** writing – review and editing, investigation. **Tong Zhang:** writing – review and editing, supervision, project administration. **Yi Jiang:** writing – review and editing, supervision, project administration.

## Disclosure

The corresponding authors, Yi Jiang and Tong Zhang, had full access to all of the data in this study and take complete responsibility for the integrity of the data and the accuracy of the data analysis.

## Ethics Statement

The data is publicly available, ethical approval was not required, and all data have been anonymized.

## Conflicts of Interest

The authors declare no conflicts of interest.

## Transparency Statement

The lead authors Yi Jiang and Tong Zhang affirm that this manuscript is an honest, accurate, and transparent account of the study being reported; that no important aspects of the study have been omitted; and that any discrepancies from the study as planned (and, if relevant, registered) have been explained.

## Supporting information


Supporting File 1

Supporting File 2

Supporting File 3

Supporting File 4

Supporting File 5

Supporting File 6

## Data Availability

The data that support the findings of this study are available in mouth ulcers‐discovery at https://data.bris.ac.uk/datasets/2j2rqgzedxlq02oqbb4vmycnc2/summary_statistics/single_trait/UKB/Raw/. These data were derived from the following resources available in the public domain: ‐ mouth ulcers‐replication, https://data.bris.ac.uk/data/dataset/459eyiulzf9y25yh6nsf550y4 ‐ leisure screen time and moderate‐to‐vigorous intensity phys, https://www.ebi.ac.uk/gwas/publications/36071172 ‐ sleep duration, https://sleep.hugeamp.org/dinspector.html?dataset=GWAS_UKBB_eu&phenotype=SleepDuration ‐ smoking heaviness and alcohol consumption, https://www.ebi.ac.uk/gwas/publications/30643251 ‐ circulating vitamin C, https://doi.org/10.2337/dc20-1328 ‐ circulating vitamin D, https://cnsgenomics.com/content/data ‐ type 2 diabetes mellitus, https://www.ebi.ac.uk/gwas/studies/GCST90018706 ‐ body mass index, metabolites and plasma proteomes, https://gwas.mrcieu.ac.uk/ ‐ circulating cytokines, https://www.ebi.ac.uk/gwas/publications/27989323 ‐ immune cells, https://www.ebi.ac.uk/gwas/publications/32929287.
