# Supplementary material for: Obesity, Lifestyle Habits and Nutrients in Relation to Oral Ulcers: A Comprehensive Mendelian Randomization Study
Source: Health Sci Rep. 2026 Jul 16;9(7):e72829. doi: 10.1002/hsr2.72829 (PMC13373697; doi:10.1002/hsr2.72829)
Supplement: Supplementary file 1 — Supporting File 1 [file HSR2-9-e72829-s002.docx]

Supplementary Fig. 1 The schematic diagram of mediation analyses.


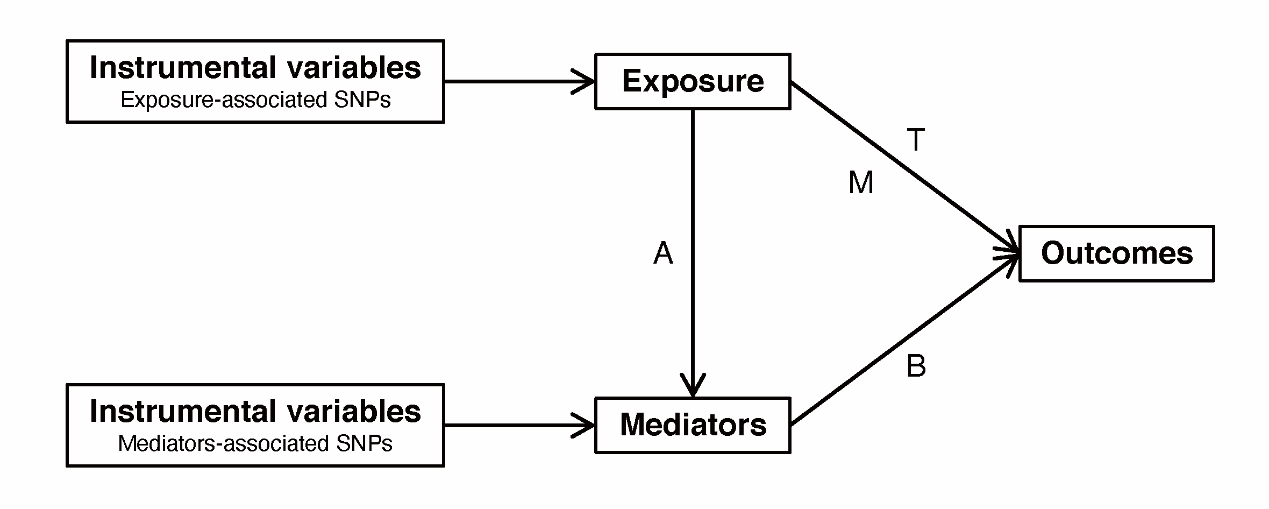


A, the effect of the exposure on the mediator; B, the effect of the mediator on the outcome; T, the total effect of exposure on the outcome. The mediation effect M is calculated by multiplying A and B, and the corresponding standard error (SE) is calculated as:

$${SE}_{M}=M\times\sqrt{\left( \frac{{SE}_{A}}{A} \right)^{2}+\left( \frac{{SE}_{B}}{B} \right)^{2}}$$

SNPs, single nucleotide polymorphisms.


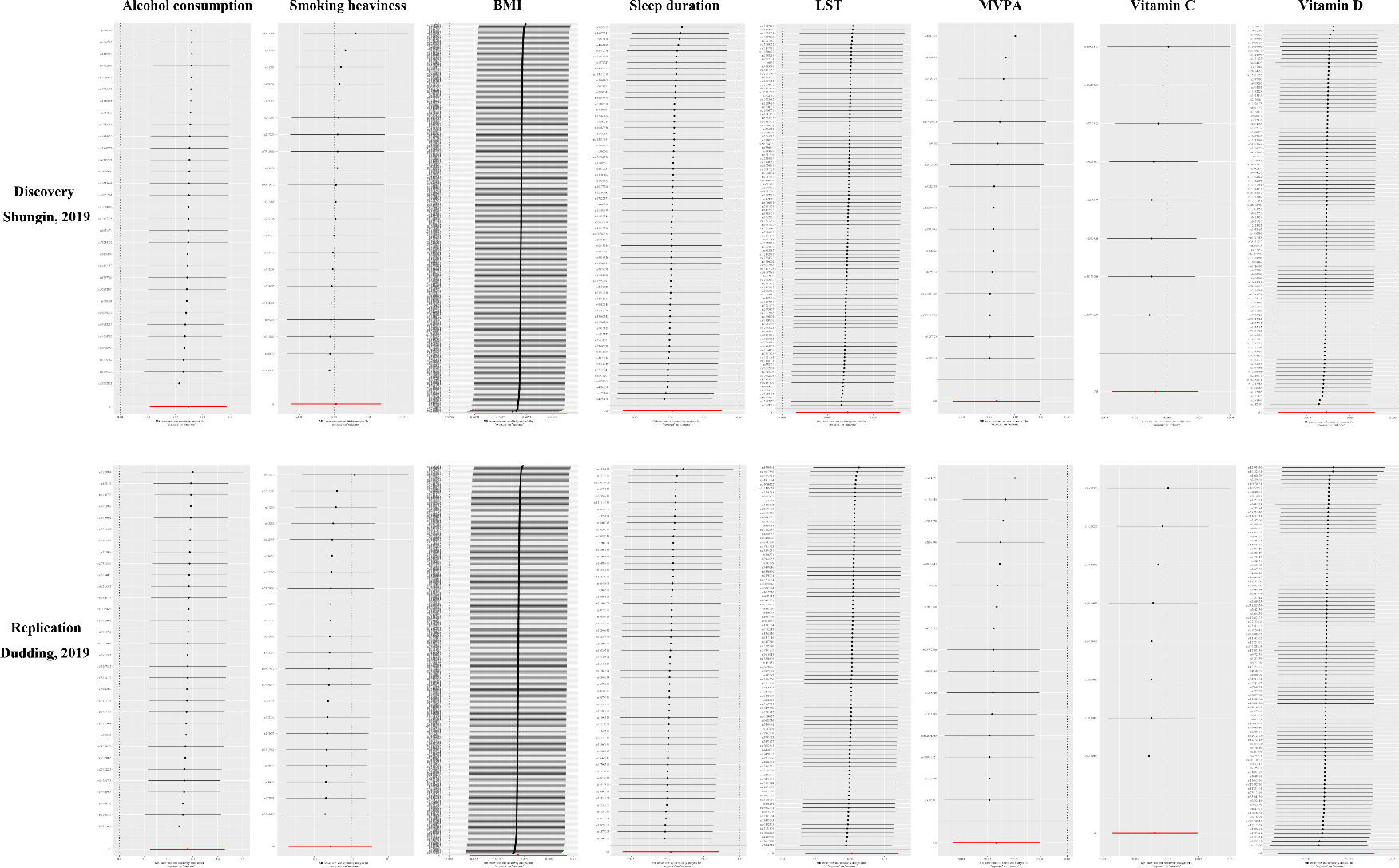


Supplementary Fig. 2 Leave one out of the causal effect of exposures on mouth ulcers

BMI, Body Mass Index; LST, Leisure Screen Time; MVPA, Moderate-to-Vigorous intensity Physical Activity


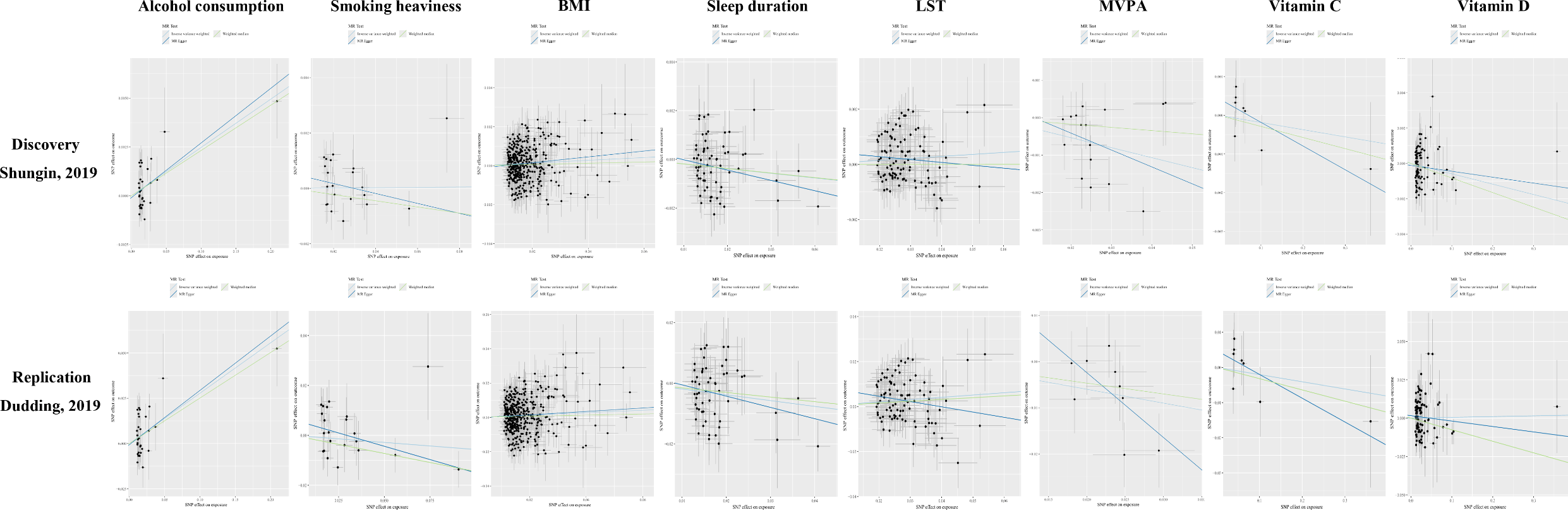


Supplementary Fig. 3 Scatter plots of the causal effect of exposures on mouth ulcers

BMI, Body Mass Index; LST, Leisure Screen Time; MVPA, Moderate-to-Vigorous intensity Physical Activity


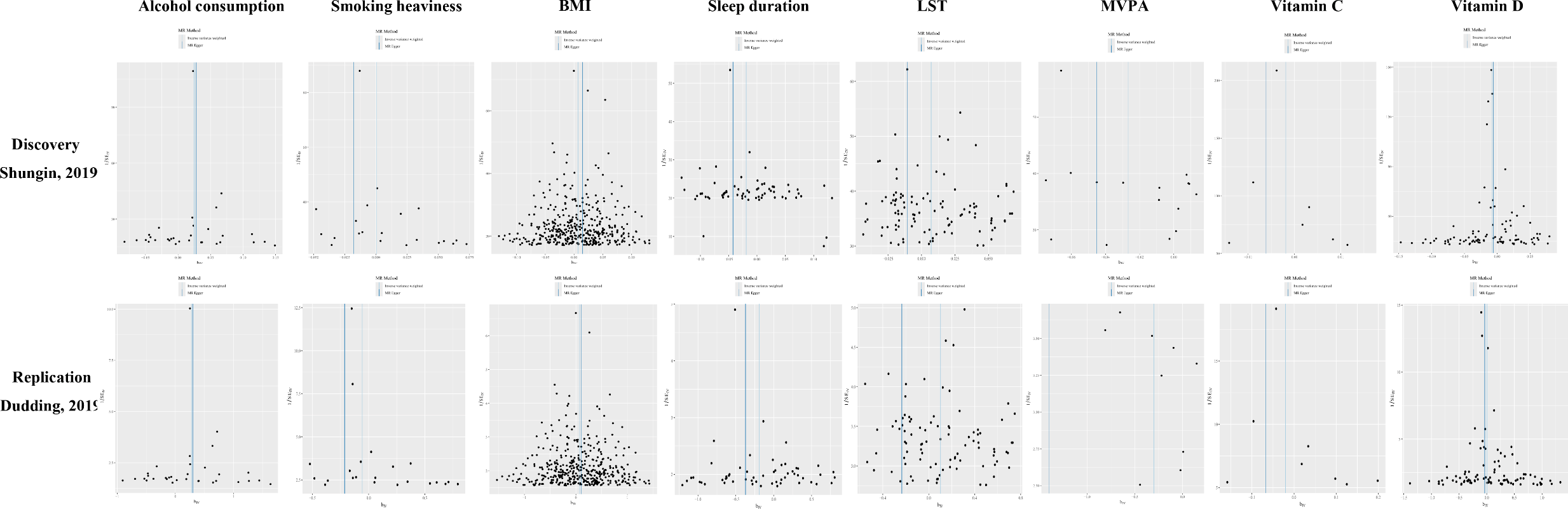
BMI, Body Mass Index; LST, Leisure Screen Time; MVPA, Moderate-to-Vigorous intensity Physical Activity

Supplementary Fig. 4 Funnel plots of the causal effect of exposures on mouth ulcers
