# Supplementary material for: Obesity, Lifestyle Habits and Nutrients in Relation to Oral Ulcers: A Comprehensive Mendelian Randomization Study
Source: Health Sci Rep. 2026 Jul 16;9(7):e72829. doi: 10.1002/hsr2.72829 (PMC13373697; doi:10.1002/hsr2.72829)
Supplement: Supplementary file 6 — Supporting File 6 [file HSR2-9-e72829-s001.docx]

Supplementary Table 1 The sensitivity analysis for UVMR included Cochran's Q test, MR-Egger intercept and MR PRESSO

|  | **Discovery (Shungin et al., 2019)** | | | | | | **Replication (Dudding et al., 2019)** | | | | | |
| --- | --- | --- | --- | --- | --- | --- | --- | --- | --- | --- | --- | --- |
|  | **Cochran's Q test** | | **MR-Egger intercept** | | **MR PRESSO** | | **Cochran's Q test** | | **MR-Egger intercept** | | **MR PRESSO** | |
|  | **Q** | **P Value** | **Intercept** | **P Value** | **RSSobs** | **P Value** | **Q** | **P Value** | **Intercept** | **P Value** | **RSSobs** | **P Value** |
| **Alcohol consumption** | 40.24 | 0.0800 | -9.43e-5 | 0.6886 | 42.27 | 0.1311 | 42.07 | 0.0887 | -6.30e-4 | 0.8050 | 43.94 | 0.1252 |
| **Smoking heaviness** | 23.40 | 0.270 | 5.35e-4 | 0.1949 | 26.37 | 0.2748 | 25.75 | 0.2161 | 6.27e-3 | 0.0574 | 29.49 | 0.2203 |
| **BMI** | 526.31 | 0.0004 | -1.46e-4 | 0.2437 | 519.37 | 0.0004 | 533.45 | 0.0002 | -4.83e-4 | 0.7296 | 531.23 | 0.0004 |
| **Sleep duration** | 123.71 | 0.0637 | 4.87e-4 | 0.1854 | 128.27 | 0.0630 | 62.66 | 0.2231 | 3.20e-3 | 0.4428 | 65.20 | 0.2214 |
| **LST** | 106.95 | 0.0005 | 4.08e-4 | 0.3677 | 67.58 | 0.4366 | 113.90 | 0.0525 | 9.27e-3 | 0.0281 | 116.52 | 0.0543 |
| **MVPA** | 23.50 | 0.0741 | 4.93e-4 | 0.5984 | 27.99 | 0.0698 | 9.18 | 0.3277 | 2.57e-2 | 0.1693 | 12.00 | 0.3466 |
| **Vitamin C** | 4.02 | 0.7770 | 4.31e-4 | 0.3248 | 5.15 | 0.8021 | 4.13 | 0.7648 | 4.76e-3 | 0.3190 | 5.29 | 0.7892 |
| **Vitamin D** | 119.05 | 0.0555 | -1.11e-4 | 0.3898 | 121.08 | 0.0597 | 114.74 | 0.0544 | 1.65e-3 | 0.2828 | 117.64 | 0.0535 |

Abbreviations: MR, Mendelian randomization; Rssobs, observed residual sum of squares. BMI, Body Mass Index; LST, Leisure Screen Time; MVPA, Moderate-to-Vigorous intensity Physical Activity

Supplementary Table 2 The sensitivity analysis for MVMR included Cochran's Q test and MVMR-Egger intercept

| **Taxa** | **Alcohol Consumption** | **Smoking heaviness** | **BMI** | **Sleep duration** | **LST** | **MVPA** | **Vitamin C** | **Vitamin D** |
| --- | --- | --- | --- | --- | --- | --- | --- | --- |
| **Covariate: Alcohol, BMI, Smoking, T2DM** | | | | | | | | |
| **Cochran's Q test** | | | | | | | | |
| **Q** | 584.69 | 584.69 | 584.69 | 578.51 | 594.83 | 569.00 | 581.53 | 564.81 |
| **P Value** | 1.07e-6 | 1.07e-6 | 1.07e-6 | 1.56e-6 | 2.19e-07 | 5.53e-6 | 1.66e-6 | 3.31e-6 |
| **MVMR-Egger intercept** | | | | | | | | |
| **Intercept** | -1.65e-4 | -1.65e-4 | -1.65e-4 | -9.91e-05 | -8.49e-05 | -8.55e-5 | -9.63e-5 | -7.93e-5 |
| **P Value** | 0.142 | 0.142 | 0.142 | 0.389 | 0.077 | 0.072 | 0.063 | 0.164 |

Abbreviations: MR, Mendelian randomization; BMI, Body Mass Index; LST, Leisure Screen Time; MVPA, Moderate-to-Vigorous intensity Physical Activity
